# Participatory video from a distance: co-producing knowledge during the COVID-19 pandemic using smartphones

**DOI:** 10.1177/14687941211038171

**Published:** 2021-08-20

**Authors:** Sonja Marzi

**Affiliations:** The London School of Economics and Political Science, UK

**Keywords:** Participatory video, co-production research, urban challenges, Colombia, collaborative research, COVID-19, digital research methods, smartphones

## Abstract

In this paper, I outline an innovative remote participatory video (PV) methodology that makes use of participants’ smartphones. It was developed as an alternative to co-production research and can be employed when face-to-face contact is impossible or undesirable. Because of the COVID-19 pandemic, face-to-face research interactions have been disrupted or become impossible. Yet it is vital to reach those who are most affected by emergencies and to include their voices. The research reported here was a collaboration between women in Medellín, Colombia, and a team of filmmakers and researchers. We developed an innovative remote PV methodology using participants’ smartphones, researching how women from poorer neighbourhoods were affected by the pandemic in their everyday lives. Here, I reflect on the strengths and weaknesses of the remote PV methodology, arguing that it offers new avenues for participants to take control of the filming and editing process, and builds technical skills and capacities that have value beyond the timeframe of the project. I conclude that the remote PV method has great potential as a stand-alone method, moving the landscape of co-production research away from a requirement for geographical co-presence and potentially shifting power and ownership towards local co-researchers and participants.

## Introduction

The COVID-19 pandemic has disrupted face-to-face research projects worldwide, with restrictions on travel and social contact introduced to avoid further health risks. Research using qualitative methodologies had to stop either immediately before or during fieldwork activities, leaving researchers not knowing when they would be able to resume their research. Even more affected has been co-produced research that relies heavily on the co-presence of researcher and participants. Nonetheless, research has not become less important and is sometimes even more pressing, especially when co-production research methodologies used with marginalised communities still have the potential to contribute to beneficial social change (Campbell et al., 2016; Kindon et al., 2007b; Pain et al., 2011). This new fieldwork landscape leads many researchers to seek alternative ways to start or continue their data collection and sometimes prompts them to change previously planned research designs (Howlett, 2021). One solution in this new fieldwork landscape, and which still permits the co-production of knowledge at a time of upheaval, is the use of smartphones to collect and share audio and visual material as well as written data.

This article emerges from a research project called ‘*Reinventada*: the realities of women in Medellín during the pandemic’, which started in May 2020 and investigates how women in poorer neighbourhoods in Medellín, Colombia, are affected by the COVID-19 pandemic in their everyday lives. Our participatory research methodology, like that of many others, relied on substantial co-presence of researcher and researched in the field. We had planned our research as a face-to-face participatory video (PV) project, whose purpose would be to make a film with women that would address their negotiation of the right to the city in Medellín. In response to the disruption brought about by the COVID-19 pandemic, however, we developed an innovative remote PV method using participants’ smartphones. The introduction of restrictions on travel and social contact globally in February and March 2020 meant that we had to cancel our original fieldwork plans and replace previously planned co-present interactions in the field with remote interactions and new research structures and practices. Since then, we have conducted the research remotely, shifted PV workshops into an online space and decided to focus on the impact of the pandemic on the women’s lives in order to amplify the voices of those who are rarely heard in emergency settings (Bradshaw, 2015; Harman, 2016).

Conducting a PV research project remotely using smartphones and digital platforms bridges two methodologies: first, (remote) digital data collection using smartphones (Do and Yamagata-Lynch, 2017; García et al., 2016; Plowman and Stevenson, 2012; Sugie, 2018), and second, participatory action research to co-produce knowledge for impact and social change (Cahill, 2007; Holt et al., 2019; Kindon et al., 2007a; Pain, 2003; Pain et al., 2011). In this paper, I provide insights about how we, a team of filmmakers and researchers based in London and Colombia, developed the remote PV method in collaboration with Colombian women participants by using digital tools, and how we at the same time followed action research principles of collaboration, education, action and reflection to co-produce knowledge (Kindon et al., 2007b; Pain, 2014; Pain et al., 2011).

I first outline the method itself and explain how we developed and employed it. Second, I reflect on the strengths and weaknesses of the remote PV method in comparison to traditional co-production PV methods. Done remotely, PV offers new avenues for participants to take control of the filming and editing process and enables them to build important technical skills and capacities that have value beyond the timeframe of the project. Yet issues of rapport and trust, and providing guidance, facilitation and training for participants are still relevant. I argue that with careful planning and reflection on the research process, the remote PV method has potential as a stand-alone method for co-producing valuable knowledge and to flatten power relationships by shifting the landscape of co-production methods away from the requirement that researcher and participants be present in the same geographical space.

## The use of smartphones in qualitative research

Mobile devices with video capability, such as smartphones, can provide a viable solution to the challenge of how to explore participants’ everyday lives during the pandemic remotely. They enable audio-visual and textual data to be collected locally and shared online between participants, researchers and practitioners. Smartphones provide new opportunities to gain access to participants *in situ* and in ‘real time’ without the co-presence of researcher and researched in one geographical space (García et al., 2016; Kaufmann, 2019). However, even though smartphones offer great remote collaboration potential, their use in co-production research approaches is still underdeveloped.

So far, the potential of smartphones for remote data collection has been recognised in research that struggles to connect with mobile groups of people, such as migrants (Kaufmann, 2019), research that wishes to reduce the influence of the researcher over and during the research process (García et al., 2016; Nash and Moore, 2018) and research during which the presence of the researcher would invade private spaces (Plowman and Stevenson, 2012). Recent studies, for instance, have used mobile phones for qualitative research projects that investigate participants’ experiences of and perspectives on their everyday lives, revealing the growing potential of smartphones as research instruments. For example, Nash and Moore (2018) asked participants to produce daily video diaries on a device of their choice, including smartphones, as part of a mixed-method study examining the impact of a women’s leadership programme on participants who were in a remote location in the Antarctic. Other studies used smartphones for app-based research and data collection, either through developing an app or through the use of existing apps such as WhatsApp that allow data to be collected in accordance with research aims (Do and Yamagata-Lynch, 2017; García et al., 2016; Hadfield-Hill and Zara, 2018; Kaufmann and Peil, 2019). Moreover, smartphone technology has been used for storytelling (Rouhani, 2019), to investigate young people’s nightlife activities (Truong et al., 2020) and to investigate children’s everyday play habits (Plowman and Stevenson, 2012).

Whilst these studies demonstrate the potential of digital technologies, and in particular smartphones, for qualitative research, they lack a collaborative approach that goes beyond the data-collection phase of the research. Noteworthy exceptions are a participatory research study by Hadfield-Hill and Zara (2018) that took into account children’s feedback when developing an app for investigating children’s daily mobility patterns in India, and studies that use mobile technology and PV methodologies in cellphilm projects (MacEntee et al., 2016; Mitchell et al., 2016). Still, the use of smartphones in co-production research that involves participants in the whole research process, from the development of the research question to the dissemination of the results (Pain et al., 2011) is limited. Most importantly, however, none of the above studies were able to create purely remote research designs. Preparation phases, such as the introduction of the research topic and the training of the participants, depended heavily on the co-presence of the researcher and the researched, something that becomes especially difficult in transnational research during health emergencies.

Nevertheless, widespread ownership of smartphones and new developments in mobile filmmaking have the potential to change PV research practices by challenging the linearity, and the division of labour, expertise and power in the production and presentation of collaborative films (MacEntee et al., 2016; Schleser, 2012: 397). Our remote PV method makes use of the technological advancement of smartphones, which can now be used to record high-quality video and connect to the internet, which opens up new avenues for including participants in the co-production process of PV, even from afar.

## Participatory video for co-production of knowledge

The growing affordability of and access to digital video technologies have led to a steady increase in the use of PV as a research methodology (Blazek and Hraňová, 2012; Milne et al., 2012). PV involves a script-less video process, directed by a group of participants, in iterative cycles of shooting and reviewing the video material to create films about participants’ experiences, needs and perspectives (Johansson et al., 1999 cited in Kindon, 2003; Lunch and Lunch, 2006).

Producing a film collaboratively opens spaces of engagement, participation, and dialogical teaching and learning which generate a process similar to Freire’s (1997) pedagogy of conscientisation (Mistry and Berardi, 2012; Vélez-Torres, 2013). It forefronts the voices of those who are systematically excluded in the knowledge production process and encourages participants to reflect on and negotiate their realities in democratic spaces of dialogue (Kindon, 2003; Pearce, 2010). PV, therefore, follows action research principles closely (Kindon et al., 2007a), rejects top-down knowledge extraction and aims to flatten traditional power hierarchies and relationships between researcher and participants (Kindon, 2003). The research process is often messy and unpredictable in its outcomes and requires the researcher to be flexible enough to follow its organic cyclical pathway (Cahill, 2007; Pearce, 2010). The co-produced film then presents a topic that has been agreed on through a dialogical approach of negotiation, which follows principles of collaboration, education, action and reflection for social change (Brickell and Garrett, 2013; Kindon et al., 2007b), with the film serving as a dissemination and advocacy instrument (Lunch and Lunch, 2006). Consequently, PV activities centre on knowledge exchange and impact, emphasising action that will improve the lives of the research participants and ease their struggles (Evans, 2016; Mistry et al., 2016; Pain et al., 2011).

Recent critiques of PV, however, have stressed that equalising power relationships between researcher and researched presents its own challenges, and while PV offers great potential to include the voices of those who often remain unheard, power relationships between researcher and researched are frequently unbalanced (Kindon, 2003). For example, pressure to satisfy funder and academic output requirements frequently shape the research process. As a consequence, researchers may interfere with the filming and editing processes for the final film and dominate the dissemination activities, which raises important questions about the ownership of the video material and about the PV process as a whole (Mistry and Berardi, 2012; Mistry et al., 2016; Wheeler, 2009). Additionally, the fact that video equipment is typically owned and provided by researchers and practitioners but is taken away again after the research ends raises important ethical and impact issues (Mitchell et al., 2016). These critiques suggest a mismatch between the theory and practice of co-production research, reinforcing traditional researcher–researched hierarchies (Cahill, 2007; Shaw, 2016) that the idea and ideal of PV is intended to challenge.

Despite these critiques, the growing potential of mobile devices with video-recording capabilities provides better opportunities to work with participants non-textually, limiting the power of the researcher to reduce the representation of participants’ voices by editing text. Direct participation and the nature of audio-visual data allow participants to express what they want to communicate more directly, and give those who feel uncomfortable with dominant academic textual methods a way of participating fully (Beebeejaun et al., 2013; Mistry et al., 2016; Rouhani, 2019). However, recent PV projects that had already started to collect data by using smartphones still depended heavily on the co-presence of researcher and researched during recruitment, training and facilitation activities (MacEntee et al., 2016). For example, the PV study by Mitchell et al. (2016) with South African teachers using cell phones to produce videos still depended on substantial guidance and skills training before, during and after the filming process. Thus, while better access, greater affordability and technical advancement have increased the use of PV as a research method, a great deal of time and energy is still required to train participants in the skills needed to produce a film collaboratively. Training is needed in basic filming and editing skills, and both researchers and participants should be trained in group facilitation skills, in order to encourage equal participation during video-making workshops (Wheeler, 2009). In addition, if PV is to become an effective tool for involving those who are most powerless and traditionally excluded, it requires a foundation of strong relationships between researcher and participants. For that reason, PV is typically based on intensive researcher–participant engagement and interaction, with face-to-face sessions involving filming and socialising, as well as mentoring of participants during workshops on collaborative analysis and editing of the video material. During the COVID-19 pandemic, this has not been possible. Using smartphones for remote PV offers new ways to engage participants in the process of making videos, and potentially to build participants’ (and researchers’) confidence, skills and capacities. In the following section I illustrate how we shifted our PV project to an online space.

## The research project

The PV project was funded by the LSE’s Knowledge Exchange and Impact (KEI) fund. The initial plan was to create a traditional face-to-face co-production research design using participatory filming in summer 2020. Before the pandemic hit, the research was going to investigate aspects of women’s right to the city in Medellín, Colombia. PV was chosen as a method because, in addition to the capacity that is built through the process of making the film, the film itself is a tangible end product for participants to own and offers the possibility of impact through dissemination activities and screenings of the film locally, and internationally online (Mitchell and Sommer, 2016). I had planned to use PV to co-produce knowledge with women in cycles of co-collecting, editing and analysing the video material, followed by workshop discussions about the dissemination of the film. However, when the COVID-19 pandemic struck, international travel restrictions disrupted plans for all face-to-face activities, and the PV project plans had to be quickly changed to a purely remote research design (Marzi, 2020). At the same time, while the initial topic of the participatory film (women’s right to the city) was still important, it became even more pressing to co-produce knowledge about the women’s everyday realities during the pandemic; in that regard, women are among the groups most affected by the pandemic (Kinyanjui, 2020; Wenham et al., 2020), and by disasters and emergencies in general (Bradshaw, 2015; Harman, 2016). In the end, the research project was named by the women participants themselves – as ‘*reinventada*’ – and explores how the pandemic affects women in Medellin who live in poor neighbourhoods. Rather than only focusing on their challenges, the women decided they wished to highlight the positive aspects of the pandemic as well, namely what they learnt during these difficult times and how they reinvented themselves. Participant-led content resulted in interesting research findings about the women’s lives, such as how important it was to them to have community mutual aid systems in their neighbourhood and access to green spaces. However, discussing the video content in detail would be beyond the scope of this paper.

We recruited 12 women in three *comunas*^1^ in Medellín. The majority of women are heads of household and mothers, and work in the informal economy. With the help of my research assistant in Medellín, I was able to reconnect with five women with whom I had already established trusting relationships during face-to-face fieldwork activities in 2019. We continued with snowball sampling, using WhatsApp and telephone calls to contact local women known to the first five. In Colombia, WhatsApp is free of charge, which makes it the main communication tool. The neighbourhoods the women live in are located on the slopes of the city and characterised by economic disadvantage and poor mobility and transport options. At the time the pandemic hit Colombia, Medellín introduced strict lockdown measures, during which inhabitants were only allowed to leave their houses on a rota system based on the numbers of people’s ID cards. This affected not only the women’s mobility and access to food and health services but also their mental health, as they were now largely confined to small houses or flats. All the women were compensated for their time, even though at the time of joining the project they were not told about the compensation, to avoid money being their primary incentive. Compensating the women for their time meant that they did not have to find other ways of generating income and decreased the risk of infection with the virus.

The project started officially in May 2020 and spanned 10 months, which included training, filming, editing activities, and the dissemination of the final film. We had initially planned for a timeframe of 15 weeks for the filming and editing activities but quickly realised that the PV process would take substantially more time in an online environment. All the women who participated owned a smartphone, but as they tended only to use their phones for WhatsApp, most of them did not know how to use any other apps or the email function. We provided internet packages remotely for participants’ smartphones and created a WhatsApp group for all the women and research team members. This group became our main communication platform and a space for women to put questions both to each other and to the research team. Additionally, we had weekly online workshops for training, editing and project-related discussions.

Although I initiated this project as principal investigator and reflect in this paper on the methodological innovation, the remote PV method was constructed in partnership with filmmakers. I only had limited PV experience, and for this reason I had already planned to work with ‘Spectacle’, an award-winning and community-focused film production company, for the initial face-to-face PV phase. Spectacle draw on decades of experience in participatory filming. Their knowledge and expertise were invaluable, especially for the training sessions for participants and the online participatory editing process. In addition to Spectacle, the research team consisted of an anthropologically trained Colombian research assistant, with whom I had already worked in 2019, and a Colombian award-winning documentary filmmaker. Both helped to guide and facilitate the PV process.

## Ethics and risk assessment

Ethical review was conducted by the LSE before the project started. However, when we changed to a remote design, we had to think innovatively about risk assessment and receiving informed consent. To receive informed consent from the women, we decided to communicate through video dialogues, which at the same time introduced the women to creating and using audio-visual data. First, we created a video that explained the project, the research process, and the role of participants (cf. for a similar approach Hammond and Cooper, 2011). We explained that selected videos produced by the women would become publicly available as short films and as part of a final documentary. In the second step we asked the participating women to share short videos, filmed themselves – so-called ‘talking heads’ – in which they agreed to participate and confirmed they had understood the information about the project, particularly its ethical aspects. Additionally, we shared the project information in text form in the WhatsApp group.

Using visual participatory methods in Medellín, and especially video, raises risk assessment challenges with respect to crime and violence (Abello-Colak and Pearce, 2015). Like Velez-Torres (2013), who used video as a method in her study in Colombia, I was concerned about the security of the women when filming, because of levels of crime and violence in their neighbourhoods. Moreover, the pandemic increased my concerns for the women’s safety, given the additional health risks and the danger of infection by the virus. Public health and lockdown measures limited women’s mobility outside their homes until the end of July 2020, and physical distancing measures meant that the women often had to film videos alone rather than in teams. Therefore, we discussed the risks and agreed filming guidelines collectively during online workshops. These included rules around (a) the consent of those being filmed, especially when filming minors, and (b) safety during filming activities, particularly in areas where showing a smartphone publicly can put women in danger of robbery, as well as guidelines on how women could avoid infection by the virus.

## Co-producing a film remotely: challenges and benefits

Translating our PV research design into a purely digital and remote one required us to rethink how we would manage and negotiate the intensive interactions required for the training, filming and editing. As with other PV projects (Mistry et al., 2015; Mitchell et al., 2016; Wheeler, 2009), there was a need to start this project with workshops allowing concentrated researcher and participant interaction to define what the co-produced film would be about and what the women wished to communicate. At the same time, we needed to create training exercises that would enable the women to learn basic filming skills using smartphones to produce short, high-quality videos that could become one screenable documentary film. We paid special attention to the development of the co-editing process of the film with the women. Editing is often dominated by filmmakers (Lunch and Lunch, 2006; Parr, 2007; Vélez-Torres, 2013) and/or done collaboratively in one room in front of one screen. Remotely, however, we had to find innovative ways of creating this one screen for collaborative editing activities in an online environment and to ensure that we shifted the selection of the videos for the final film into the hands of the women. While we are aware that our approach of PV still reinforces Western ideas of how a documentary is filmed and edited by default (Kindon, 2016), we were determined to challenge the traditional power relationships as much as possible that usually feature in film-editing processes and to prevent ‘hierarchical power relations’ from creating ‘distanced or unreflexive claims to knowledge’ by making a film collaboratively *alongside* the women (Kindon 2003: 143).

**Figure 1.**
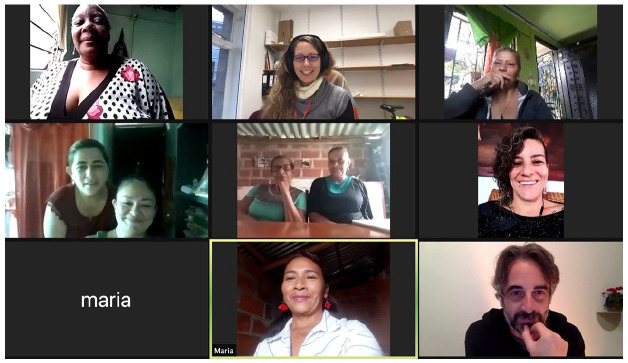
Screenshot of Zoom meeting.

One of the aims of this research was skill development and capacity-building (for participants and researchers), and technical and filming skills were a part of the skillset we aimed to build. Training, filming and editing were done in cycles of action and reflection. We started by providing filming instructions, and training on filming in weekly online sessions (see Figure 1), where we discussed filming techniques and how to send videos via e-mail. The initial training sessions followed a design similar to the one discussed by Lunch and Lunch (2006) and contained guided exercises on filming ‘first shots’. The women would send their videos online, we shared these as a video compilation through an online link and re-watched them with the group to improve their filming techniques. The sharing of videos online also meant that the women could delete the videos, instead of storing them on their phones. Once the women had a basic filming knowledge, we used the weekly workshops to play back the footage they had filmed and to discuss the video material. Filmmakers led the training sessions on filming and editing, and provided the technical support, while the researchers facilitated the action and reflection process with a focus on discussions on the content of the film. Finally, during the editing process, the filmmakers created video compilations of the filmed material that would contain a timecode, which women used to identify the video sections they wanted to include into the final film. During the weekly online editing workshops, we would play back the selected compilations to decide collectively on the material for the final film and to see if any more filming was needed to communicate women’s realities during the pandemic in the way they wanted.

I never thought we would do all we did! Looking back, I realised all these shots we took are a way of showing we are part of this city during the pandemic [. . .] Me, becoming a film director, and making a film? I never imagined that. (D., *comuna* 3)

Using smartphones to produce short videos remotely and combine them into a final documentary provided an opportunity to collect accounts of women’s realities during the pandemic, while at the same time, the weekly online workshops were a space for knowledge exchange, learning and democratic dialogue and discussion. However, as with other studies mentioned (Kindon, 2003; Mistry et al., 2016; Vélez-Torres, 2013), doing PV is resource-intensive and time-consuming, and requires long-term engagement and excellent facilitation skills, something that we realised was even more of a challenge when conducting PV remotely. Rather than having compressed time of 5 days a week, 8 h a day, together, for several weeks in a row, as we would have had if we’d been filming together in person, shifting online meant that the time available for training, discussion and editing was reduced to one weekly online meeting of 2.5 h. As a consequence, the online PV space required us to become more flexible with the total length of the project and with the number of hours spent both on- and offline working to ensure that training and editing needs were met.

Despite the time-management challenges for us, the research team, for the women participants the shift to the remote design was actually more beneficial. The women in this study were very enthusiastic about learning how to film with their smartphones so that they could communicate their needs, the challenges they faced and the things they had learnt about how to manage their everyday lives during the pandemic. The remote nature of the method allowed them to do this on their own terms and to fit it around their other responsibilities. Shooting videos independently provided them with the ability to participate more flexibly.

Nevertheless, the reduced time of one 2.5-h workshop a week came at a cost in terms of action and reflection on the video content and material. Running out of time each week, we had to cut discussions of the video material short so that we had enough time for training and to make editing decisions for the final documentary. Thus, especially in terms of space for reflection on the material produced, which is a crucial part of co-production and action research, I was rather dissatisfied and wished we had had more time and space for in-depth discussion of some of the topics that the women had included in the film. Hence, at times it was challenging to have a greater focus on the PV process rather than on the end product. However, finishing the film was a priority, both for the women and for us, because we all wanted to show the women’s realities during the pandemic, and funding requirements limited our timeframe.

As with a study by Cunsolo Willox et al. (2013), which used digital cameras for storytelling, we did not require women to have any prior knowledge or experience with digital infrastructure. We found that women had different levels of familiarity with the digital world. We used a variety of training techniques and engaged in one-to-one sessions with participants to check that all participants felt comfortable shooting videos and sharing them for discussion in online meetings. Particular challenges that created some frustration among participants in the first few weeks of the project included sending video material of varying sizes through an app, by email and via other cloud systems, and joining online meetings with smartphones. All those problems were solved with practice and repetition, during which we learned collaboratively how best to use the different technological platforms. Once they were solved, we all felt an immense sense of achievement, and the women started to feel a sense of ownership and confidence from their new skills. Moreover, as the pandemic prevented women from living their offline lives as they used to, they increasingly acknowledged the value of learning extra digital skills and saw how they could use them outside the project, an effect of PV that has also been reported in other studies (Shaw, 2016).

One of the most daunting technological challenges was the participatory editing process. Usually done together in one room and in front of one screen, it allows participants to play around with different video sequences themselves and create a rough cut of the final film. As described above, we had to change this process and provide women with video compilations tagged by timecodes so that they could choose the material that they wanted to include into the film. Wheeler (2009: 15) argues that the nature of digital video editing makes it practically impossible to make this process participatory. Technical difficulties need overcoming, which requires commitment and engagement from participants. This is even more the case when developing an editing process online. Nevertheless, we were committed to making the editing of the film as much of a collaborative process as the filming and discussions. And while we were not able to sit together in one room, we were still able to select the videos for the final film together in one online space on one virtually shared screen. It took 3 weeks until the women felt comfortable with the process of selecting timecodes before the weekly online workshops. Because we had watched most of the video footage already, during online sessions in previous weeks, there was not much disagreement over its content. In fact, the opposite was the case, and the women found it difficult to let go of some of the video material to reduce the final film^2^ to 32 min in length to ensure participation and visibility of the whole group (Lomax et al., 2011: 238).

In contrast to more traditional forms of collaborative filmmaking, we did not use storyboards or develop a linear storytelling process before the filming and editing started (MacEntee et al., 2016; Mitchell et al., 2016). Instead, we developed the storyline throughout the editing process, with repeated cycles of editing and reflecting on film content and its order, during which women would choose and reshuffle content. Additionally, the filmmakers from Spectacle offered extra editing workshops, which women could join for several hours a day two or three times a week if they wanted to. We then re-watched updated versions of the film in weekly online workshops for approval by all women participants. Only the very last version of the film was edited by the filmmakers alone, and it was then re-watched by the women so that they could approve it and in case they wanted any final changes made. We as a research team would only support the process through moderation and facilitation of content discussions and would leave any decision about film content to the women themselves.

## Negotiating power, trust and control remotely

The videos shot by the women in this project touch upon inequalities that existed before the pandemic as well as the increased challenges the women have struggled with since it started. For example, the former included inequalities within the city, extreme poverty among their neighbours, income insecurity and economic hardship, while the latter included topics such as food insecurity, poor (mental) health, decreased access to healthcare, and the changes the pandemic has caused in their homes and neighbourhoods. Nevertheless, the women did not only want to show the challenges and struggles caused by the pandemic, but more importantly they wanted to highlight how they learnt from the hardship it created for them; how they reinvented themselves. Building a space of trust, action and reflection was crucial for enabling women to raise these issues through film.

Mondays are a space that really helps you a lot because there are face-to-face virtual hugs, because even though we are so distant I think they are face-to-face . . . (A., *comuna* 3)

Mistry et al. (2015) describe in their own PV study how crucial the co-presence of the researchers in the field was – in providing advice, guidance and feedback – for building trusting relationships and confidence of participants and with the research team. While we were not able to be geographically co-present, we agreed that intense interactions between the research team and the women were necessary to build rapport and trust. One of the most effective means of creating this rapport and trust was the weekly online meetings. The continuity for the women (and us) made the PV workshops a routine part of the week. Especially at the peak of the pandemic, this space was welcomed by everyone involved because it provided a sense of purpose while they were confined at home. Additionally, the women created their own space for weekly reflection. A couple of weeks into the project, they decided to start 30 min before the official project meeting, so that they could talk about non-project-related topics. They felt it would give them back a sense of normality if they could have the sort of conversations in this online space which would typically take place on the streets. Follow-up interviews further revealed that these 30 min of normal conversation alleviated their mental health problems by creating a sense of solidarity between them. The weekly meetings actually became such an important part of their week that when we tried to change the timing, it was impossible, as all the women organised their lives around this particular meeting. Hence the women created a sense of community through the filming activities and routine meetings, during which they realised they shared experiences of injustice, such as inequality in the city, limited access to healthcare and agreement on the importance of green spaces, and that they also used similar mechanisms to cope with the additional insecurity caused by the pandemic.

As the project progressed, the women became increasingly committed and engaged in the process. To support the participatory ethos that aimed to shift power relations and gaze (Kindon, 2003), we decided to introduce role-swapping (Shaw, 2016) during workshops as part of the production and editing process. Thus, the women chaired the weekly online workshops, decided which themes and content were selected and took a leading part in the editing process. While role-swapping can be used to coerce rather than encourage, if the researcher uses their power to get participants to take over leading roles (Shaw, 2016), in our project it actually reduced some confrontational group dynamics, as some participants had felt that more vocal women were dominating the weekly workshops. At the same time, the fact that the women filmed their videos independently and the move to a remote space also offered additional opportunities for women to take control over the PV process. In our remote project, not physically being in the place where the film was shot reduced my knowledge of what the film could cover, for example. Apart from my suggestion that the film might cover the realities of the women during the pandemic, the themes were all suggested and developed by the women themselves. For example, it was the women who decided the film should highlight how they reinvented themselves in response to the challenges caused by the pandemic. We embraced this dynamic of ownership further by introducing professional film production vocabulary. We called the women ‘*directoras de la pelicula*’ (film directors) and explained that we would only be able to work according to their instructions, namely on the selection of videos for the final film. From this moment onwards, and in follow-up interviews, the women would actually refer to themselves as film directors and protagonists, demonstrating how they became owners of the process and their film.

While I, in my role as academic researcher, found it frequently unsettling to lose control over the filming process and the content of the film, the fact that the women filmed videos on their own devices independently and without supervision by the research team was giving them a degree of control and ownership that would probably not have been possible otherwise. As Western researchers, we tend to control our research by travelling to field sites, moderating and facilitating our research and data collection, and making sure we are able to produce the academic outcomes required by funders and the audit culture of universities (Evans, 2016). I was not able to completely eliminate those power imbalances that are intensified by academic and funding requirements. In our remote PV project, however, I had only limited control over the film content, which meant that power over the filming process was indeed shifted to patricipants. Yet, at the same time, ethical issues arise that need careful reflection during the filming process and the re-watching of video material, especially with regard to blurring the boundaries of the women’s private and public spaces.

## Conclusion: the potential of remote PV as a stand-alone method

In this paper, I have sought to set out an innovative methodological tool for conducting PV remotely in a manner that is rewarding from the point of view of both process and outcome. Prompted by the COVID-19 pandemic, we decided to embrace the potential of smartphones and online platforms to replace face-to-face interactions in the field with remote relations, technology and new research structures and practices. In describing the remote PV method here, I have demonstrated the feasibility of remote PV and shown that it can function as a valid and workable research method, capable of transmitting and amplifying participants’ voices, building their skills, and producing a rewarding end product. Moreover, I have explained how the PV process, which traditionally relies on intensive in-person interaction, can be shifted – through remote relationships mediated by videos, text interactions, and online meetings, and using different techniques and structures – away from a requirement that researcher and participants are co-present.

However, the remote PV process does not come without its challenges. Having read accounts of other PV research (Kindon, 2003; Mistry et al., 2016), we first thought that training, facilitation, guidance and editing would become our most demanding tasks when it was not possible to spend compressed time together in one geographical space, but we realised that it was actually the change in the total time spent working on the project that required us to adapt most. The reduction of participatory filming and editing time to weekly online workshops lasting 2.5 h required us to extend the research timeframe ‘in the field’ from 6 weeks to over 10 months. However, what seemed to be the biggest adjustment for us as a research team turned out to be one of the greatest benefits in terms of time and research design for the women. Being able to organise the filming around their other responsibilities, especially during the pandemic in 2020, allowed them to participate more comfortably; although special attention needs to be paid to ethical issues that arise, blurring the boundaries of the women’s public and private spaces. Additionally, the editing process for the final film allowed the women to embrace their role as film directors and to take ownership of the film content. Though, the reduction in time spent together also reduced our space for action and reflection. Our role, by contrast, shifted from technical and research guidance to that of a facilitator for the filming and editing process – navigating the technological platform and taking instructions from the participants. This shift of ownership, while not able to eliminate hegemonic power completely, flattened traditional hierarchies between the research team and the participants.

Cunsolo Willox et al. (2013) argue:

Our task and our responsibility as social science researchers, is to continue to search for and work with methods that ‘produce, reveal, and enable’ the voices, stories, and lived experiences of the participants in as unhindered and uninhibited manner as possible.

I hope I have demonstrated that remote PV offers exactly that and opens new avenues for participants to take greater control of the filming and editing process. At the same time, participants build important technical skills and capacities that have value beyond the timeframe of the project. Moreover, the heightened confidence developed by the women through the process reinforced the ethos of inclusive research, which Holt et al. (2019) describe as both co-productive and emancipatory for those often unheard.

Despite the challenges of conducting the PV project remotely – especially with regard to issues that relate to the reduced time to reflect on the content of the video material – I consider this innovative method to be a way forward in altering the co-production landscape that potentially can shift hegemonic power towards local co-researchers and participants. While challenges are still present, I argue that PV conducted remotely offers new avenues for co-production research that are more difficult to access when research team and participants are co-present for shorter, compressed timeframes. The way that our women participants became committed to the project and took control of the process without the geographical co-presence of researcher and participants shows that remote PV can be a valid method in itself, one that could fruitfully be explored further in the future to develop co-production methods that challenge hegemonic power through collaboration and are applicable during emergencies when the co-presence of researchers and participants is impossible.

## Notes

1 Medellin is divided in 16 *comunas.*

2 You can watch the trailer of the final documentary here: https://vimeo.com/554445666

## References

[bibr1-14687941211038171] Abello-Colak A Pearce J (2015) Securing the global city?: an analysis of the ‘Medellín Model’ through participatory research. Conflict, Security & Development 15: 197–228.

[bibr2-14687941211038171] Beebeejaun Y Durose C Rees J , et al. (2013) ‘Beyond text’: exploring ethos and method in co-producing research with communities. Community Development Journal 49: 37–53.

[bibr3-14687941211038171] Blazek M Hraňová P (2012) Emerging relationships and diverse motivations and benefits in participatory video with young people. Children’s Geographies 10: 151–168.

[bibr4-14687941211038171] Bradshaw S (2015) Engendering development and disasters. Disasters 39: 54–75. 10.1111/disa.12111 25494957

[bibr5-14687941211038171] Brickell K Garrett BL (2013) Geography, film and exploration: women and amateur filmmaking in the Himalayas. Transactions of the Institute of British Geographers 38: 7–11.

[bibr6-14687941211038171] Cahill C (2007) The personal is political: developing new subjectivities through participatory action research. Gender, Place & Culture 14: 267–292.

[bibr7-14687941211038171] Campbell H Vanderhoven D with Chatterton P , et al. (2016) Knowledge that Matters: Realising the Potential of Co-production. Manchester, NH: N8 Research Partnership.

[bibr8-14687941211038171] Cunsolo Willox A Harper SL Edge VL (2013) Storytelling in a digital age: digital storytelling as an emerging narrative method for preserving and promoting indigenous oral wisdom. Qualitative Research, 13: 127–147.

[bibr9-14687941211038171] Do J Yamagata-Lynch LC (2017) Designing and developing cell phone applications for qualitative research. Qualitative Inquiry 23: 757–767.

[bibr10-14687941211038171] Evans R (2016) Achieving and evidencing research ‘impact’? Tensions and dilemmas from an ethic of care perspective. Area 48: 213–221.

[bibr11-14687941211038171] Freire P (1997) Pedagogy of the Oppressed. New York: Continuum.

[bibr12-14687941211038171] García B Welford J Smith B (2016) Using a smartphone app in qualitative research: the good, the bad and the ugly. Qualitative Research 16: 508–525.

[bibr13-14687941211038171] Hadfield-Hill S Zara C (2018) Being participatory through the use of app-based research tools. In: Carter B Coyne I (eds) Being Participatory: Researching with Children and Young People. Cham: Springer International Publishing, 147–169.

[bibr14-14687941211038171] Hammond SP Cooper NJ (2011) Participant information clips: a role for digital video technologies to recruit, inform and debrief research participants and disseminate research findings. International Journal of Social Research Methodology 14: 259–270.

[bibr15-14687941211038171] Harman S (2016) Ebola, gender and conspicuously invisible women in global health governance. Third World Quarterly 37: 524–541.

[bibr16-14687941211038171] Holt L Jeffries J Hall E , et al. (2019) Geographies of co-production: learning from inclusive research approaches at the margins. Area 51: 390–395.

[bibr17-14687941211038171] Howlett M (2021) Looking at the ‘field’ through a Zoom lens: methodological reflections on conducting online research during a global pandemic. Qualitative Research 1–16. 10.1177/1468794120985691 PMC9095994 35663097

[bibr18-14687941211038171] Kaufmann K (2019) Mobile methods: doing migration research with the help of smartphones. In: Smets K Leurs K Georgiou M , et al. (eds) The SAGE Handbook of Media and Migration. London, UK: SAGE.

[bibr19-14687941211038171] Kaufmann K Peil C (2019) The mobile instant messaging interview (MIMI): using WhatsApp to enhance self-reporting and explore media usage in situ. Mobile Media & Communication 8: 229–246.

[bibr20-14687941211038171] Kindon S (2003) Participatory video in geographic research: a feminist practice of looking? Area 35: 142–153.

[bibr21-14687941211038171] Kindon S (2016) Participatory video as a feminist practice of looking: ‘take two!’. Area 48: 496–503.

[bibr22-14687941211038171] Kindon S Pain R Kesby M (eds) (2007a) Participatory Action Research Approaches and Methods: Connecting People, Participation and Place. Abingdon: Routledge.

[bibr23-14687941211038171] Kindon S Pain R Kesby M (2007b) Participatory action research: origins, approaches and methods. In: Kindon S Pain R Kesby M (eds) Participatory Action Research Approaches and Methods: Connecting People, Participation and Place. Abingdon: Routledge.

[bibr24-14687941211038171] Kinyanjui N (2020) COVID-19: a double burden for women in conflict settings. The LSE Women, Peace and Security blog.

[bibr25-14687941211038171] Lomax H Fink J Singh N , et al. (2011) The politics of performance: methodological challenges of researching children’s experiences of childhood through the lens of participatory video. International Journal of Social Research Methodology 14: 231–243.

[bibr26-14687941211038171] Lunch N Lunch C (2006) Insights into Participatory Video: A Handbook for the Field, InsightShare.

[bibr27-14687941211038171] MacEntee K Burkholder C Schwab-Cartas J (eds) (2016) What’s a Cellphilm?: Integrating Mobile Phone Technology into Participatory Visual Research and Activism. Rotterdam: Sense. 10.1080/17441692.2016.1169307 29932852

[bibr28-14687941211038171] Marzi S (2020) Conducting transnational participatory research with women during covid-19 remotely: An impossibility? The LSE International Development blog. Available at: https://blogs.lse.ac.uk/internationaldevelopment/2020/05/22/conducting-transnational-participatory-research-with-women-during-covid-19-remotel…1/4

[bibr29-14687941211038171] Milne E-J Mitchell C Lange N (2012) Handbook of Participatory Video. Lanham, MD: AltaMira Press.

[bibr30-14687941211038171] Mistry J Berardi A (2012) The challenges and opportunities of participatory video in geographical research: exploring collaboration with indigenous communities in the North Rupununi, Guyana. Area 44: 110–116.

[bibr31-14687941211038171] Mistry J Berardi A Bignante E , et al. (2015) Between a rock and a hard place: ethical dilemmas of local community facilitators doing participatory research projects. Geoforum 61: 27–35.

[bibr32-14687941211038171] Mistry J Bignante E Berardi A (2016) Why are we doing it? Exploring participant motivations within a participatory video project. Area 48: 412–418.

[bibr33-14687941211038171] Mitchell CM Sommer M (2016) Participatory visual methodologies in global public health. Global Public Health 11: 521–527. 27105078 10.1080/17441692.2016.1170184

[bibr34-14687941211038171] Mitchell C De Lange N Moletsane R (2016) Me and my cellphone: constructing change from the inside through cellphilms and participatory video in a rural community. Area 48: 435–441.

[bibr35-14687941211038171] Nash M Moore R (2018) Exploring methodological challenges of using participant-produced digital video diaries in Antarctica. Sociological Research Online 23: 589–605.

[bibr36-14687941211038171] Pain R (2003) Social geography: on actionorientated research. Progress in Human Geography 27: 649–657.

[bibr37-14687941211038171] Pain R (2014) Impact: striking a blow or walking together? ACME: An International Journal for Critical Geographies 13: 19–23.

[bibr38-14687941211038171] Pain R Kesby M Askins K (2011) Geographies of impact: power, participation and potential. Area 43: 183–188.

[bibr39-14687941211038171] Parr H (2007) Collaborative film-making as process, method and text in mental health research. Cultural Geographies 14: 114–138.

[bibr40-14687941211038171] Pearce J (2010) Co-producing knowledge critical reflections on researching participation. In: Pearce J (ed.), Participation and Democracy in the Twenty-First Century City: Non-Governmental Public Action. London: Palgrave Macmillan.

[bibr41-14687941211038171] Plowman L Stevenson O (2012) Using mobile phone diaries to explore children’s everyday lives. Childhood 19: 539–553.

[bibr42-14687941211038171] Rouhani L (2019) Using digital storytelling as a source of empowerment for rural women in Benin. Gender & Development 27: 573–586.

[bibr43-14687941211038171] Schleser M (2012) Collaborative mobile phone filmmaking. In: Milne E-J Mitchell C DE Lange N (eds) Handbook of Participatory Video. Lanham, MD: AltaMira Press.

[bibr44-14687941211038171] Shaw J (2016) Emergent ethics in participatory video: negotiating the inherent tensions as group processes evolve. Area 48: 419–426.

[bibr45-14687941211038171] Sugie NF (2018) Utilizing smartphones to study disadvantaged and hard-to-reach groups. Sociological Methods & Research 47: 458–491.

[bibr46-14687941211038171] Truong J Labhart F Santani D , et al. (2020) The emotional entanglements of smartphones in the field: on emotional discomfort, power relations, and research ethics. Area 52: 81–88.

[bibr47-14687941211038171] Vélez-Torres I (2013) Reflections on a participatory documentary process: constructing territorial histories of dispossession among Afro-descendant youth in Colombia. Area 45: 299–306.

[bibr48-14687941211038171] Wenham C Smith J Morgan R (2020) COVID-19: the gendered impacts of the outbreak. The Lancet 395: 846–848. 10.1016/S0140-6736(20)30526-2 PMC7124625 32151325

[bibr49-14687941211038171] Wheeler J (2009) The life that we don’t want: using participatory video in researching violence. IDS Bulletin, 40.

